# Identification of *NRAS* Diagnostic Biomarkers and Drug Targets for Endometrial Cancer—An Integrated in Silico Approach

**DOI:** 10.3390/ijms232214285

**Published:** 2022-11-18

**Authors:** Larsen Alessandro, Kat-Jun Eric Low, Aisha Abushelaibi, Swee-Hua Erin Lim, Wan-Hee Cheng, Sook-keng Chang, Kok-Song Lai, Yap Wai Sum, Sathiya Maran

**Affiliations:** 1 Department of Biotechnology, Faculty of Applied Sciences, UCSI University, Kuala Lumpur 56000, Malaysia; 2 Advanced Membrane Technology Research Centre (AMTEC), School of Chemical and Energy Engineering (FCEE), Universiti Teknologi Malaysia, Johor 81310, Malaysia; 3 Health Sciences Division, Abu Dhabi Women’s College, Higher Colleges of Technology, Abu Dhabi P.O. Box 25026, United Arab Emirates; 4 Faculty of Health and Life Sciences, INTI International University, Persiaran Perdana BBN, Putra Nilai, Nilai 71800, Malaysia; 5 He & Ni Academy, Office Tower B, Northpoint Mid Valley City, Kuala Lumpur 59200, Malaysia; 6 School of Pharmacy, Monash University Malaysia, Subang Jaya 47500, Malaysia

**Keywords:** endometrial carcinoma, diagnostic markers, prognostic gene, protein–ligand interactions, *NRAS* gene

## Abstract

The diagnosis of endometrial cancer involves sequential, invasive tests to assess the thickness of the endometrium by a transvaginal ultrasound scan. In 6–33% of cases, endometrial biopsy results in inadequate tissue for a conclusive pathological diagnosis and 6% of postmenopausal women with non-diagnostic specimens are later discovered to have severe endometrial lesions. Thus, identifying diagnostic biomarkers could offer a non-invasive diagnosis for community or home-based triage of symptomatic or asymptomatic women. Herein, this study identified high-risk pathogenic nsSNPs in the NRAS gene. The nsSNPs of NRAS were retrieved from the NCBI database. PROVEAN, SIFT, PolyPhen-2, SNPs&GO, PhD-SNP and PANTHER were used to predict the pathogenicity of the nsSNPs. Eleven nsSNPs were identified as “damaging”, and further stability analysis using I-Mutant 2.0 and MutPred 2 indicated eight nsSNPs to cause decreased stability (DDG scores < −0.5). Post-translational modification and protein–protein interactions (PPI) analysis showed putative phosphorylation sites. The PPI network indicated a GFR-MAPK signalling pathway with higher node degrees that were further evaluated for drug targets. The P34L, G12C and Y64D showed significantly lower binding affinity towards GTP than wild-type. Furthermore, the Kaplan–Meier bioinformatics analyses indicated that the NRAS gene deregulation affected the overall survival rate of patients with endometrial cancer, leading to prognostic significance. Findings from this could be considered novel diagnostic and therapeutic markers.

## 1. Introduction

Endometrial cancer (EC) is the sixth most frequent malignancy among women, and the cases were reported to be 417,000 in 2020 [1]. The incidence has increased by 132% over the past 30 years, attributed to increased risk factors, predominantly obesity and population aging [2]. Women from low- and middle-income countries (LMICs) are more likely to die from endometrial cancer than those from high-income countries (HICs) due to poor access to medical care and a higher proportion of aggressive, non-endometrioid tumors diagnosis [3]. Seventy percent of patients with atypical perimenopausal or postmenopausal vaginal bleeding are diagnosed early, allowing for timely management [4]. However, there are cases diagnosed at an advanced stage, thus decreasing proper treatment. The different stages of EC significantly influence the prognosis; in stage I, the five-year survival rate is approximately 95%, whereas, in stage IV, it is only approximately 14% [5]. Thus, the identification of diagnostic biomarkers for early diagnosis is important, especially in high-risk individuals with extreme obesity, diabetes, hypertension, and Lynch syndrome. 

The RAS family is extensively studied in cancer research. RAS consists of KRAS, NRAS and HRAS, and mutations in these genes have been frequently reported in colorectal cancer, pancreatic ductal adenocarcinoma, lung adenocarcinoma, melanoma and some hematological cancers [6]. 

NRAS gene is a well-known driver oncogene, and mutations in NRAS are reported to cause a poor response to anti-EGFR targeted therapy. Mutations in several pathways have been reported to be responsible for its development. Studies have reported causative roles of mutations in the PI3K pathway, WNT signaling, RAS–RAF pathways, transcriptional regulation, DNA damage response, and FBXW7-related genes [7]. Liu and colleagues (2019) reported that estrogen receptor alpha (ERα) activates the MAPK signaling pathway to promote the development of EC [8]. Recent studies have demonstrated that the RAS/MAPK pathway is the most frequently mutated pathway in patients with cancer, with driver mutations in NRAS or KRAS occurring in 40 to 55% of newly diagnosed patients [9]. RAS and its isoforms are GTPases which will be activated when GTP binds to it, relay signals and activate some downstream pathways responsible for cell growth. Mutations in RAS lock them in an active state. Thus, there will be a continuous unstoppable downstream signaling process resulting in cancer. 

Considering the pathological role of NRAS in the RAS/MAPK pathway, its pathogenesis towards EC remains to be elucidated. We hypothesize that understanding the structural and functional effects of NRAS could shed light on discovering the diagnosis and prognosis of EC. Therefore, this study, for the first time, examines the role of nsSNPs of NRAS using bioinformatics tools in understanding its pathogenesis towards EC. This study utilized multi-level functional and structural as described in our previous study (Lim et al., 2021) in predicting the novel biomarker and elucidating the role of nsSNPs of NRAS in the RAS/MAPK pathway.

## 2. Results

### 2.1. Prediction of High-Risk Pathogenic and Stability of nsSNPs

A total of 147 nsSNPs of NRAS were extracted from the NCBI database. High-risk pathogenic nsSNPs were predicted using PROVEAN, SIFT, PolyPhen-2, PredictSNP, SNPs&GO, PANTHER and PhD-SNP. Eleven nsSNPs, including rs121913248, rs267606920, rs1465850103, rs121913250, rs121434595, rs121434596, rs1557982817, rs869025573, rs397514553, rs1308441238 and rs752508313, were predicted as high-risk pathogenic by at least six of the tools. The high-risk damaging nsSNPs were then submitted to the MutPred server to confirm the pathogenicity (Table 1). Structural stability analysis by I-Mutant2.0 (Table 2) indicated nsSNPs, including rs267606920, rs121913250, rs121434595, rs1557982817, rs869025573, rs397514553, rs1308441238 and rs752508313, cause a decrease in stability to the resultant proteins, with DDG value < −0.5, indicating its greater impact towards the proteins.

### 2.2. Identification of Post-Translational Modification (PTM) Sites

**Figure 1:**
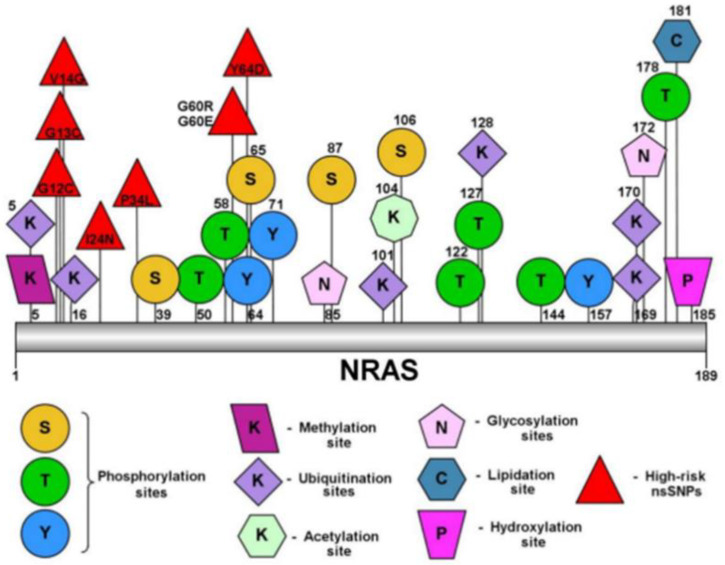
Putative PTM sites of high-risk nsSNPs of NRAS protein.

Post-translational modifications (PTM) are the process of proteins undergoing chemical modifications to become functional and participate in respective cellular activities. Putative PTM sites in the NRAS proteins and the eight high-risk pathogenic nsSNPs were predicted using BDM-PUB, NetPhos-3.1 and MusiteDeep. BDM-PUB predicted six ubiquitination sites on lysine residues of NRAS protein (K5, K16, K101, K128, K169, K170), while NetPhos-3.1 predicted twelve sites of phosphorylation which occurred on all three possible amino acids namely lysine, threonine and tyrosine (S106, S65, S87, T122, T127, T144, T178, T50, T58, Y157, Y64, Y71). MusiteDeep predicted only one site of each methylation (K5), lipidation (C181), hydroxylation (P185) and acetylation (K104), followed by two sites of glycosylation that happened in asparagine residues of positions 85 and 172. Figure 1 shows the putative PTM sites.

### 2.3. Protein–Protein Interaction (PPI) and Molecular Network Analysis

**Figure 2:**
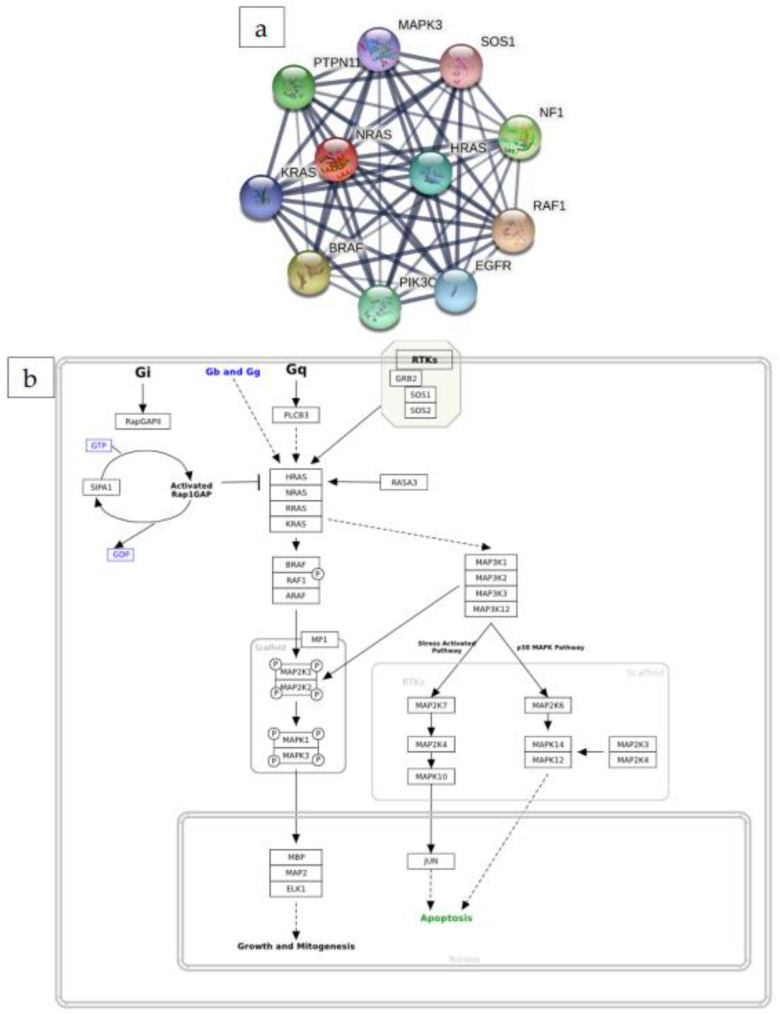
PPI and molecular network analysis. (**a**) The interaction of NRAs with ten partners, and (**b**) the molecular network analysis.

PPI showed that NRAS interacts with RAF1, BRAF, NF1, PTPN11, PIK3CA, HRAS, EGFR, KRAS, MAPK3, and SOS1 proteins (Figure 2a). Molecular networks showed that the NRAS gene is involved in MAPK cascade pathways involving apoptosis, cell growth, cell proliferation and cell cycle (Figure 2b).

### 2.4. Prognosis of NRS in EC Malignancy

**Figure 3:**
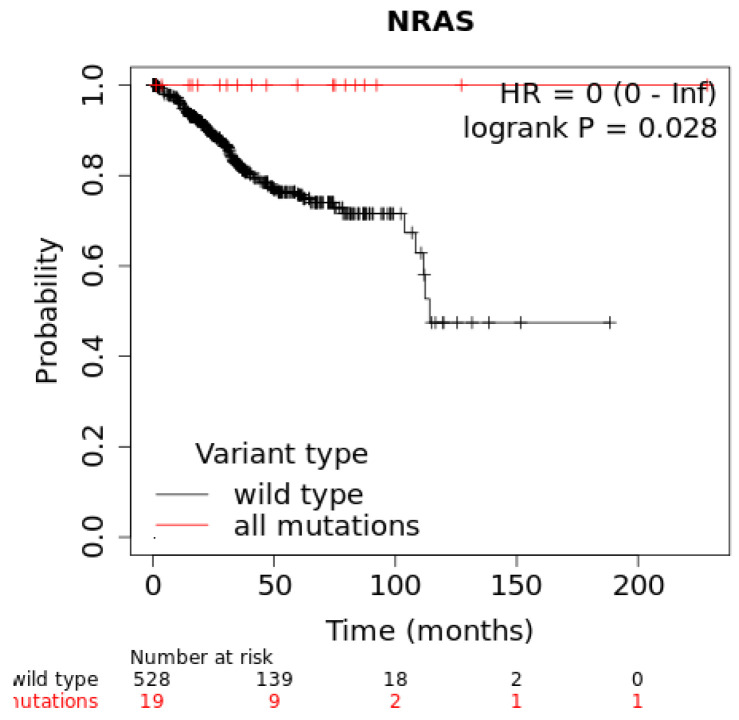
Kaplan–Meier plot showing the correlation between the deregulation of NRAS and overall survival rate EC.

A Kaplan–Meier plotter was used to determine the prognostic value of the NRAS gene by combining gene expression and EC cancer patient survival. The analysis showed a hazard ratio (HR) = 0 and logrank *p*-value = 0.028 for EC cancer (Figure 3).

### 2.5. Prediction of Structural Alteration of NRAS nsSNPs

**Figure 4:**
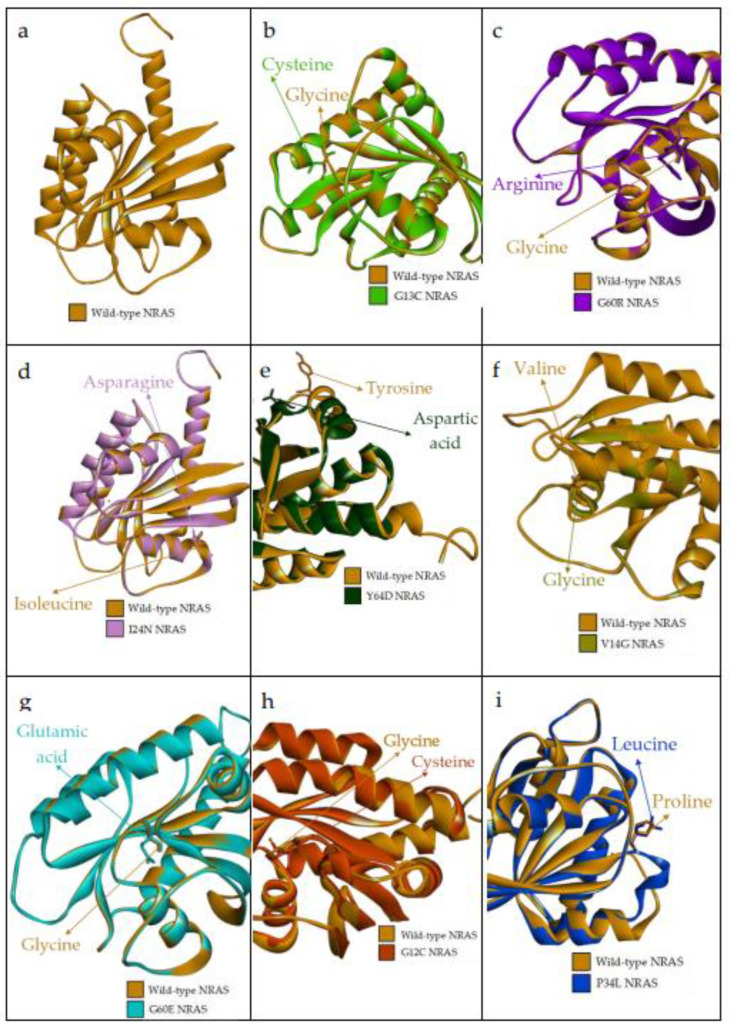
3D protein structure of superimposed wild-type NRAS and its mutated protein predicted by SWISS-MODEL. (**a**) The 3D structure of wild-type NRAS protein; (**b**) superimposed structure of wild-type NRAS protein with mutated I24N NRAS protein; (**c**) superimposed structure of wild-type NRAS protein with mutated G60E NRAS protein; (**d**) superimposed structure of wild-type NRAS protein with mutated G13C NRAS protein; (**e**) superimposed structure of wild-type NRAS protein with mutated Y64D NRAS protein; (**f**) superimposed structure of wild-type NRAS protein with mutated V14G NRAS protein; (**g**) superimposed structure of wild-type NRAS protein with mutated G60R NRAS protein; (**h**) superimposed structure of wild-type NRAS protein with mutated G12C NRAS protein; (**i**) superimposed structure of wild-type NRAS protein with mutated P34L NRAS protein.

As hydrophobicity has a significant contribution to protein function and structure, the hydrophobicity of wild-type and mutant residues were analyzed in SWISS-Model to investigate their physicochemical properties. All the predicted high-risk nsSNPs showed a hydrophobic to hydrophilic conversion except for rs397514553 and rs1308441238. Hence, mutant 3D models of nsSNPs, rs267606920, rs121913250, rs121434595, rs1557982817, rs869025573, and rs752508313, localized in the NRAS domain and predicted high-risk pathogenic with structural change from hydrophobic to hydrophilic were modeled using SWISS-Model (Figure 4). 

### 2.6. NRAS Protein Docking

NRAS proteins were individually docked with GTP using the AutoDock Vina (ver 1.1.2). Protein docking was carried out to the changes and compare the binding affinity of the GTP to wild-type NRAS protein and the mutated NRAS protein. The results were generated in kcal/mol with negative numbers indicating greater binding affinity. Binding affinity refers to the strength of binding interaction between protein to its ligand; the smaller the value, the lesser energy required by the protein and ligand to bind to each other. 

**Figure 5:**
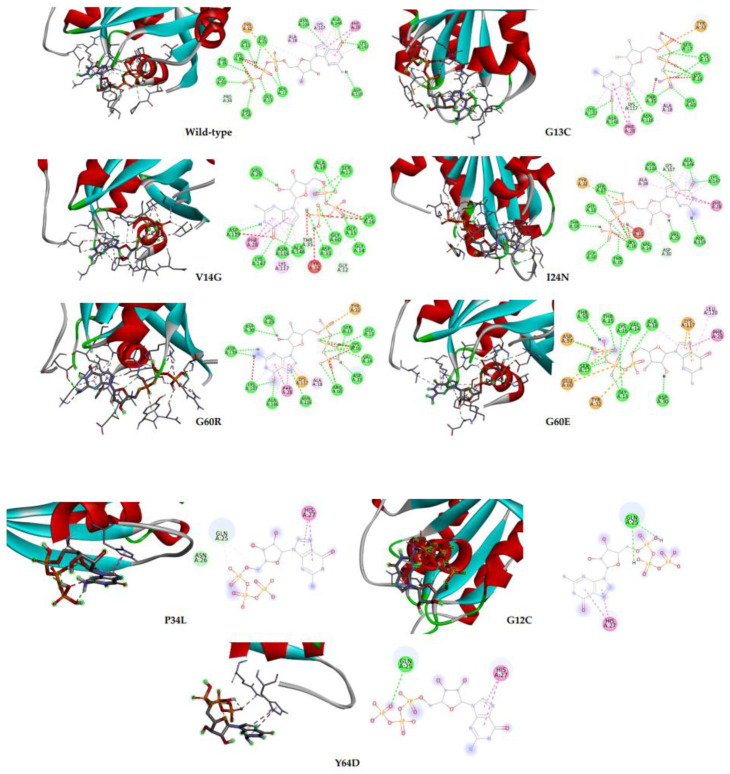
Docking profile of NRAS protein (wild-type and mutated) and GTP. The right panel shows the 2D representation of the interaction with ligands and the receptors in the binding pocket.

Table 3 shows the binding affinity between both wild-type and mutated NRAS and GTP as ligands. The rs121913250 (G12C), rs397514553 (P34L) and rs752508313 (Y64D) showed negative values of binding affinity; however, the degree of spontaneousness is not as high as the wild-type NRAS. Hydrogen bonds have the most influence in stabilizing protein and its ligand binding and predominantly contribute to the specificity of molecular recognition. Hydrogen bonds surrounding the wild-type NRAS–GTP complex are Gly-13, Val-14, Gly-15, Lys-16, Ser-17, Thr-35, Thr-58, Gly-60, Asn-116, Asp-119, Ala-146, Lys-147. The G13C mutation made hydrogen bonds of Val-14, Gly-15, Thr-58 and Asp-119 disappear, while NRAS V14G lost hydrogen bonds of Gly-13, Thr-35 (changed into a carbon–hydrogen bond) and Thr-58 but formed new hydrogen bonds in Ala-18 (previously Pi-Alkyl bond), Val-29 and Asp-33. Furthermore, G60R mutation did not change/lose the hydrogen bond in position 60; however, it lost a few hydrogen bonds (Gly-13, Gly-15, Lys-16, Ser-17, Thr-35, Thr-58) and formed a few hydrogen bonds (Val-29, Asp-30, Asp-33). The Van der Waals forces only appeared in NRAS P34L, whereas Gln-25 and His-27 hydrogen bonds were the only bonds involved in binding three NRAS mutated proteins with the lowest binding affinity. Remarkably, these Gln-25 and His-27 were not involved in the wild-type NRAS–GTP complex. Figure 5 is the 2D diagram of the interaction between NRAS proteins and GTP. 

## 3. Discussion

Endometrial cancer (EC) is the most common malignancy affecting women in developed countries, and the incidence rate has increased since 2000 [10]. EC, which occurs mostly in developed countries, is commonly carcinoma instead of sarcoma [7]. EC originates from the uterine epithelium, and epidemiologically, EC is related to diabetes, obesity, late menopause and increasing age. Additionally, Lynch Syndrome, Polymerase Proofreading Associated Polyposis, and Cowden Syndrome increase the risk of EC genetically [7]. The two most common pathways responsible for apoptosis, cell growth, proliferation and differentiation, are highly involved in endometrial cancer: the PI3K/Akt and MAPK pathways [11]. The MAPK pathway involves three main kinases, namely MAP3K, MAP2K and MAPK. They activate the cascade and phosphorylate downstream proteins [12]. 

The NRAS gene and its family KRAS and HRAS have been explained to be related to different types of cancers [13]. NRAS are the prevalent oncogenes contributing 16–25% among all cancers [14]. The study also reported that mutated NRAS affects melanoma. NRAS is mostly involved in the mitogen-activated protein kinase (MAPK) pathway and the phosphoinositide 3-kinase (PI3K)/protein kinase B (AKT) cascade. These two pathways/cascades are responsible for cell proliferation, survival, differentiation and apoptosis. Therefore, mutations in the NRAS gene would disrupt these pathways and result in uncontrollable cell growth. Unlike Out of 147 nsSNPs extracted from NCBI, 11 nsSNPs; rs121913248 (A18P), rs267606920 (G60E), rs1465850103 (D57N), rs121913250 (G12C), rs121434595 (G13C), rs121434596 (G13V), rs1557982817 (G60R), rs869025573 (I24N), rs397514553 (P34L), rs1308441238 (V14G), and rs752508313 (Y64D) were predicted as high-risk deleterious. Further stability analysis predicted rs267606920, rs121913250, rs121434595, rs1557982817, rs869025573, rs397514553, rs1308441238 and rs752508313 nsSNPs to be pathogenic and to decrease stability of the proteins. Destabilized protein may cause protein degradation, improper folding/misfolding and eventually cause diseases such as neurodegenerative diseases and genetic disorders [15]. 

Post-translational modifications analysis was carried out to determine modification of the side chain of amino acids of proteins, which may affect protein structure and functions and disrupt biological processes [16]. BDM-PUB predicted ubiquitination sites of NRAS are in positions 5, 16, 101, 128, 169 and 170. Campbell and Philips (2021) reported that NRAS had a few PTM sites of C118 for nitrosylation, K42 for sumoylation and K5 for ubiquitination. Furthermore, the ubiquitination of NRAS has an adverse effect, reducing MAPK signaling. Twelve phosphorylation sites consisting of T50, T58, T122, T127, T144, T178, Y64, Y71, Y157, S65, S87 and S106 were predicted in NRAS, which is in agreement with a study by Yin and colleagues, who showed that NRAS was activated by phosphorylation on S89 by STK19 [17]. Methylation (K5), glycosylation (N85 and N172), lipidation (C181), hydroxylation (P185) and acetylation (K104) sites of NRAS were also predicted. The lipidation of C181 was also predicted by Ahearn et al. (2012). Palmitoylation (lipidation) is necessary for the NRAS protein trafficking from the endomembrane system to the plasma membrane and is involved in the transformation of NRAS-driven myeloid [18,19].

Protein–protein interactions showed the connections between RAF1, BRAF, HRAS, KRAS, MAPK3, SOS1, EGFR, NF1, PTPN11, and PIK3CA. RAF1 and BRAF are members of the RAF family and strongly interact with NRAS in the MAPK cascade (Figure 2b). The role of RAF proteins in NRAS-driven melanoma was discussed by Dorard and colleagues [20]. RAF/MAPK is a main downstream effector of oncogenic RAS in melanoma. Figure 2b also shows that SOS1 and MAPK3 protein were directly related to the activation of NRAS. It is reported that mutations in SOS1 and MAPK3 could lead to the over-activation of RAS pathway (including NRAS), which results in lung adenocarcinoma and melanoma [21,22]. PI3K/Akt/mTOR and MAPK pathways are constitutively activated by phosphorylation [23]. However, the data regarding biomarkers of the two pathways have not really been discovered.

The Kaplan–Meier plot was used to assess the correlation between the expression of the NRAS gene and survival in different types of tumors. The analysis showed that mutation in the NRAS gene would significantly affect the survival of patients having uterine corpus endometrial carcinoma (*p*-value = 0.0282). NRAS mutation, specifically G12V, is present as a mutation hotspot in endometrial cancer [24]. However, NRAS mutation generally contributes only 5% to endometrial carcinoma [25]. Although the occurrence is rare, NRAS mutation consistently happens in type I tumors whenever the RAS–MAPK pathway gets activated. Moreover, a genotyping assessment showed that mutations in NRAS exclusively happened in uterine-origin tumors [26]. NRAS mutation (e.g., Q61K) has also been identified in low-grade serous ovarian cancer [27].

Molecular recognition refers to the interaction between macromolecules with small molecules through noncovalent interactions to form a complex. There are two main characteristics: specificity and affinity [28]. Knowing protein–ligand binding affinity is useful in designing new drugs and detecting the effects of mutated protein on the binding of the ligand. Protein docking confirmed that three (P34L, G12C and Y64D) out of eight nsSNPs had significantly lower binding affinity than wild-type NRAS. While one other variant (G60E) had a slightly lower binding affinity than wild-type. The other four had a similar binding affinity. As observed in Table 3, wild-type NRAS had the greatest binding affinity with GTP. Alongside the binding affinity, Figure 5 showed that the 3 mutations with weaker binding relationships also have lesser binding sites with the GTP. As opposed to the other five NRAS proteins with more than 10 binding sites on average, the four proteins have only two binding sites each. Reduced binding affinity may affect or end the protein function [29]. 

The prognostic factors of EC are directly correlated with its mortality. Despite the stringent guidelines of The European Society for Medical Oncology (ESMO), the European Society of Gynecological Oncology (ESGO), the European Society for Radiotherapy and Oncology (ESTRO), and the European Society of Pathology (ESP) consortiums in managing diagnosis treatment and follow-ups, the mortality remains elevated [30]. Recent studies have highlighted the importance of prognostic indicators, which should be evaluated at the time of diagnosis [31]. This then leads to the importance of an accurate and timely diagnosis.

Considering the above, findings from this study pave the way toward identifying diagnostic biomarkers with potential clinical application. Furthermore, we consign confidence that these biomarkers can be applied as a prognostic factor towards determining the preoperative risk of recurrence and directing surgical treatment.

## 4. Materials and Methods

### 4.1. Retrieving SNPs

**Figure 6:**
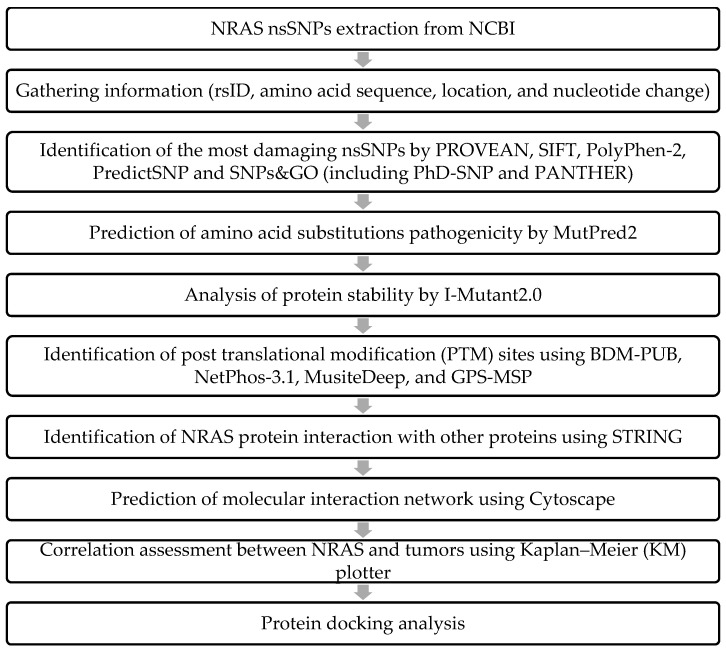
Diagrammatic representation of methodology.

The nsSNPs of NRAS were obtained from NCBI dbSNP (Gene ID: 4893) database (National Center for Biological Information) (https://www.ncbi.nlm.nih.gov/snp/ (accessed on 24 May 2022)). “NRAS” was submitted as the query and was set limited to an only missense mutation. SNP IDs, wild-type nucleotides and their variations were retrieved. Duplicates were removed before proceeding to the next step. Amino acid sequence of the NRAS gene was also retrieved from UniProt (P01111) (accessed in 24 May 2022). The methodology was conducted as previously described by Lim and colleagues [32] (Figure 6).

### 4.2. Identification of High-Risk nsSNPs

A total of seven tools were used to predict the damaging and deleterious nsSNPs of the NRAS gene; PROVEAN (Protein Variation Effect Analyzer) [http://provean.jcvi.org/index.php (accessed on 24 May 2022)] [33], SIFT (Sorting Intolerant From Tolerant) [https://sift.bii.a-star.edu.sg/ (accessed on 24 May 2022)] [34] PolyPhen-2 (Polymorphism Phenotyping v2) [http://genetics.bwh.harvard.edu/pph2/ (accessed on 24 May 2022)] [35], PredictSNP [https://loschmidt.chemi.muni.cz/predictsnp/ (accessed on 21 June 2022)] [36], SNPs&GO [https://snps.biofold.org/snps-and-go/snps-and-go.html (accessed on 29 May 2022)] [37], PhD-SNP (Predictor of human Deleterious Single Nucleotide Polymorphisms) and PANTHER (Protein ANalysis THrough Evolutionary Relationships). The cut-off score of PROVEAN is −2.5, while SIFT is 0.05. A score above those numbers was considered benign. For PolyPhen2, the score varies between 0 and 1, where 0.45 to 0.95 is considered possibly damaging and 0.95 to 1.0 probably damaging. Mutations in PredictSNP are considered deleterious by having a score interval of (0, +1>). By having a score of more than 0.5, the nsSNPs predicted by SNPs&GO, PhD-SNP and PANTHER will be reported as “Disease” with a higher score resulting in a higher reliability index. nsSNPs predicted as “Disease/Deleterious” by at least six tools were picked for further analysis.

### 4.3. Prediction of Pathogenicity of Amino Acid Substitutions

MutPred2 [http://mutpred.mutdb.org/ (accessed on 30 May 2022)] [38] was used to verify the pathogenicity of the nsSNPs predicted high-risk deleterious. The FASTA format of the protein and the mutation information were submitted. A score above 0.5 was considered pathogenicity (above 0.68 for a 10% false positive rate and above 0.8 for a 5% false positive rate).

### 4.4. Protein Stability Analysis

The stability of protein was predicted using I-Mutant2.0 [https://folding.biofold.org/i-mutant/i-mutant2.0.html (accessed on 30 May 2022)] [39]. The protein sequence was submitted, and the default setting of 25 °C temperature was at pH 7. Amino acid mutations predicted to be “decreasing” were subjected to further analysis.

### 4.5. Post Translational Modification (PTM) Sites Identification

The BDM-PUB (Prediction of Ubiquitination sites with Bayesian Discriminant Method) [http://bdmpub.biocuckoo.org/ (accessed on 30 May 2022)] [40], NetPhos-3.1 (phosphorylation of serine, threonine and tyrosine) [https://services.healthtech.dtu.dk/service.php?NetPhos-3.1 (accessed on 30 May 2022)] [41] and MusiteDeep (many types of prediction model—phosphorylation, glycosylation, ubiquitination, SUMOylation, methylation, acetylation, hydroxylation, palmitoylation and cyclization) [https://www.musite.net/ (accessed on 30 May 2022)] [42,43] were used to predict the PTM sites. The FASTA format of wild-type and mutated NRAS protein sequence were submitted.

### 4.6. Prediction of Protein-Protein Interaction and Molecular Interaction Network

The protein–protein interaction was determined using STRING [https://string-db.org/ (accessed on 1 July 2022)] [44,45]. Cytoscape [https://cytoscape.org/ (accessed on 1 July 2022)] was used to retrieve the prediction of molecular interaction networks [46]. 

### 4.7. Prognosis Analysis

A Kaplan–Meier (KM) plot was shown to assess the correlation between NRAS and cancers. The KM plotter was used to determine the prognostic *p*-value [https://kmplot.com/analysis/ (accessed on 1 July 2022)] [47]. Pan-cancer DNA was chosen, and “NRAS” was submitted as the query. The survival curves and log *p*-values were obtained. Only cancer with a log *p*-value below 0.05 was considered as significantly affected by NRAS nsSNPs.

### 4.8. Molecular Docking Analysis

Molecular docking determined whether the mutation would reduce the binding affinity between the mutated protein and the ligand. The Amino acid sequence of wild-type NRAS and all high-risk pathogenic nsSNPs was submitted to SWISS-MODEL [https://swissmodel.expasy.org/ (accessed on 11 July 2022)] for 3D structure modelling [48]. The predicted 3D structure was modelled using homology templates from the known protein structure having similar sequences with both wild-type and mutated NRAS. Simplified Molecular-Input Line-Entry System (SMILES) sequence of Guanosine Triphosphate (GTP), as the ligand of NRAS, was retrieved from PubChem [https://pubchem.ncbi.nlm.nih.gov/ (accessed on 8 August 2022)] and converted into PDB file using OPENBABEL [http://www.cheminfo.org/Chemistry/Cheminformatics/FormatConverter/index.html (accessed on 11 July 2022)] [49]. 

GTP was processed in AutoDock 4.2.6 to automatically detect the root and choose rotatable bonds, respectively [50]. AutoDock 4.2.6 was also used to remove water, add hydrogen and add Kollman charges into the protein. Protein docking was carried out with coordinates x = 18.065000, y = −0.343000 and z = 7.238000 [51]. The docking results were viewed using BIOVIA Discovery Studio Visualizer.

## 5. Conclusions

This study contributed to understanding the causative roles of high-risk pathogenic nsSNPs towards endometrial cancer. NRAS protein is involved in many cell-cycle-regulating pathways, and nsSNPs in NRAS are associated with cancer and poor prognosis of endometrial cancer, thus serving as a novel diagnostic biomarker. In addition, this study also reports structural-based evidence indicating molecular changes due to nsSNPs.

## Figures and Tables

**Table 1:** High-risk nsSNPs of NRAS predicted using PROVEAN, SIFT, PolyPhen-2, PredictSNP, SNPs&GO, PhD-SNP, PANTHER and MutPred2.

SNP ID	Amino Acid Change	PROVEAN	SIFT	PolyPhen-2	PredictSNP	SNPs&GO		PhD-SNP		PANTHER		MutPred2	
		Score	Score	Score	Score	RI	Prob	RI	Prob	RI	Prob	Score	Pred
rs121913248	A18P	−4.32	0.001	1	1 (C > G), 0.1257 (C > T)	9	0.972	8	0.915	2	0.603	0.93	Pathogenic
rs267606920	G60E	−7.49	0	1	1.0000	9	0.933	7	0.826	8	0.881	0.95	Pathogenic
rs1465850103	D57N	−4.52		0.996	1.0000	8	0.912	6	0.814	0	0.509	0.92	Pathogenic
rs121913250	G12C	−7.09		0.656	1.0000	9	0.937	8	0.881	3	0.644	0.92	Pathogenic
rs121434595	G13C	−7.72		0.999	1.0000	9	0.957	9	0.926	7	0.863	0.94	Pathogenic
rs121434596	G13V	−7.65		0.996	1.0000	9	0.963	8	0.924	6	0.78	0.93	Pathogenic
rs1557982817	G60R	−7.49		1	1.0000	8	0.923	7	0.844	8	0.91	0.96	Pathogenic
rs869025573	I24N	−5.32		1	1.0000	9	0.954	4	0.699	3	0.648	0.94	Pathogenic
rs397514553	P34L	−8.56		1	1.0000	8	0.916	3	0.641	8	0.917	0.85	Pathogenic
rs1308441238	V14G	−5.86		1	1.0000	8	0.915	7	0.84	4	0.686	0.93	Pathogenic
rs752508313	Y64D	−8.84		1	0.1578	10	0.975	8	0.91	1	0.527	0.96	Pathogenic

**Table 2:** Stability prediction of mutated NRAS protein using I-Mutant2.0.

SNP ID	Amino Acid Change	Stability	RI
rs267606920	G60E	Decrease	1
rs121913250	G12C	Decrease	5
rs121434595	G13C	Decrease	5
rs1557982817	G60R	Decrease	7
rs869025573	I24N	Decrease	7
rs397514553	P34L	Decrease	2
rs1308441238	V14G	Decrease	10
rs752508313	Y64D	Decrease	4
rs121913248	A18P	Increase	1
rs1465850103	D57N	Increase	1
rs121434596	G13V	Increase	2

**Table 3:** Binding affinity between NRAS proteins and GTP.

Protein–Ligand Complex	Amino Acid Residues Involved in NRAS–GTP Complex Stabilization	Binding Affinity (kcal/mol)
Wild-Type NRAS–GTP	Gly-13, Val-14, Gly-15, Lys-16, Ser-17, Ala-18, Phe-28, Tyr-32, Pro-34, Thr-35, Thr-58, Gly-60, Asn-116, Lys-117, Asp-119, Ala-146, Lys-147	−10.8
NRAS G13C–GTP	Cys-13, Lys-16, Ser-17, Ala-18, Phe-28, Tyr-32, Thr-35, Gly-60, Asn-116, Lys-117, Ala-146, Lys-147	−10.6
NRAS V14G–GTP	Gly-12, Gly-14, Gly-15, Lys-16, Ser-17, Ala-18, Phe-28, Val-29, Tyr-32, Asp-33, Thr-35, Gly-60, Asn-116, Lys-117, Asp-119, Ala-146, Lys-147	−10.5
NRAS I24N–GTP	Gly-13, Val-14, Gly-15, Lys-16, Ser-17, Ala-18, Phe-28, Val-29, Asp-30, Tyr-32, Thr-35, Thr-58, Gly-60, Asn-116, Lys-117, Asp-119, Ala-146, Lys-147	−10.4
NRAS G60R–GTP	Gly-13, Val-14, Lys-16, Ser-17, Ala-18, Phe-28, Val-29, Asp-30, Tyr-32, Asp-33, Arg-60, Asn-116, Lys-117, Asp-119, Ala-146, Lys-147	−10.3
NRAS G60E–GTP	Gly-13, Val-14, Lys-16, Ser-17, Ala-18, Phe-28, Asp-30, Tyr-32, Thr-35, Asp-57, Thr-58, Glu-60, Lys-117, Leu-120	−9
NRAS P34L–GTP	Gln-25, Asn-26, His-27	−2.9
NRAS G12C–GTP	Gln-25, His-27	−2.8
NRAS Y64D–GTP	Gln-25, His-27	−2.6

## Data Availability

All data are available in the manuscript.
